# Temperature-Corrected Calibration of GS3 and TEROS-12 Soil Water Content Sensors

**DOI:** 10.3390/s24030952

**Published:** 2024-02-01

**Authors:** Paolo Nasta, Francesca Coccia, Ugo Lazzaro, Heye R. Bogena, Johan A. Huisman, Benedetto Sica, Caterina Mazzitelli, Harry Vereecken, Nunzio Romano

**Affiliations:** 1 Department of Agricultural Sciences, AFBE Division, University of Naples Federico II, 80055 Portici, Italy; f.coccia.94@gmail.com (F.C.); ugolazzaro@tiscali.it (U.L.); benedetto.sica@unina.it (B.S.); caterina.mazzitelli@unina.it (C.M.); nunzio.romano@unina.it (N.R.); 2 Agrosphere Institute (IBG-3), Forschungszentrum Juelich GmbH, 52425 Juelich, Germany; h.bogena@fz-juelich.de (H.R.B.); s.huisman@fz-juelich.de (J.A.H.); h.vereecken@fz-juelich.de (H.V.)

**Keywords:** capacitance sensors, soil apparent permittivity, soil temperature, Cosmic Ray Neutron Sensor, sensor performance

## Abstract

The continuous monitoring of soil water content is commonly carried out using low-frequency capacitance sensors that require a site-specific calibration to relate sensor readings to apparent dielectric bulk permittivity (*K_b_*) and soil water content (*θ*). In fine-textured soils, the conversion of *K_b_* to *θ* is still challenging due to temperature effects on the bound water fraction associated with clay mineral surfaces, which is disregarded in factory calibrations. Here, a multi-point calibration approach accounts for temperature effects on two soils with medium to high clay content. A calibration strategy was developed using repacked soil samples in which the *K_b_*-*θ* relationship was determined for temperature (*T*) steps from 10 to 40 °C. This approach was tested using the GS3 and TEROS-12 sensors (METER Group, Inc. Pullman, WA, USA; formerly Decagon Devices). *K_b_* is influenced by *T* in both soils with contrasting *T*-*K_b_* relationships. The measured data were fitted using a linear function *θ* = *a*$\sqrt{K_{b}}$ + *b* with temperature-dependent coefficients *a* and *b*. The slope, *a*(*T*), and intercept, *b*(*T*), of the loam soil were different from the ones of the clay soil. The consideration of a temperature correction resulted in low RMSE values, ranging from 0.007 to 0.033 cm^3^ cm^−3^, which were lower than the RMSE values obtained from factory calibration (0.046 to 0.11 cm^3^ cm^−3^). However, each experiment was replicated only twice using two different sensors. Sensor-to-sensor variability effects were thus ignored in this study and will be systematically investigated in a future study. Finally, the applicability of the proposed calibration method was tested at two experimental sites. The spatial-average *θ* from a network of GS3 sensors based on the new calibration fairly agreed with the independent area-wide *θ* from the Cosmic Ray Neutron Sensor (CRNS). This study provided a temperature-corrected calibration to increase the accuracy of commercial sensors, especially under dry conditions, at two experimental sites.

## 1. Introduction

Measurements of near-surface and root-zone volumetric soil water content, *θ* (L^3^ L^−3^), are of paramount importance for understanding hydrological processes in the vadose zone. Soil water content controls the exchange and partitioning of water and energy fluxes between the land surface and the atmosphere and influences the carbon cycle, crop growth, and the fate of contaminants. In addition, soil water content is used for calibration and validation of remote sensing data and the evaluation of process-oriented, distributed eco-hydrological models [1]. The laboratory-based thermogravimetric method (or the oven-drying method) is recognized as the most accurate approach for determining *θ*, but it is destructive, time-consuming, expensive, and labor-intensive. To circumvent these drawbacks, indirect methods have gained popularity by measuring soil bulk dielectric properties that are related to *θ* [2]. In the 1980s, time domain reflectometry (TDR) emerged as an accurate technique to estimate *θ* [3,4,5,6]. The TDR technique is non-destructive, relatively rapid, and therefore more practical than the thermogravimetric method. However, a TDR set-up for automatic monitoring is limited to a relatively small area, and the costs for large-scale monitoring (>1000 m^2^) remain prohibitive.

In the last decades, many companies have developed low-cost, low-frequency (i.e., frequency < 100 MHz) sensors to monitor soil water content based on the measurement of the apparent dielectric bulk permittivity (*K_b_*). Many of these sensors also measure other soil characteristics, such as soil temperature (*T*) and bulk electrical conductivity (*EC_b_*). Capacitance sensors determine soil dielectric permittivity by measuring the charge time of a capacitor (i.e., the soil-probe system) for a given voltage. Nowadays, capacitance sensors record the soil water content digitally with low energy consumption, easy maintenance, and logging capabilities. Multiple low-cost sensors can be installed at several positions and at different soil depths and form a wireless sensor network that allows for the investigation of the spatio-temporal dynamics of soil water content at high temporal and spatial resolution [7]. 

However, due to their relatively low operating frequency, capacitance sensors are susceptible to secondary effects (e.g., induced by strong variations in soil temperature and salinity-induced electrical conductivity) that affect their measurement accuracy and precision [8,9,10,11,12,13,14,15,16]. Therefore, proper calibration is mandatory to reliably convert the soil dielectric permittivity into soil water content, especially for fine-textured soils [17,18,19,20,21,22]. 

In this study, we investigated the GS3 sensor because it is still widely used, even though it is no longer commercially available. For instance, in the Upper Alento River Catchment (southern Italy), two test sites are equipped with SoilNet wireless sensor networks controlling 80 GS3 sensors (METER Group Inc., Pullman, WA, USA; formerly Decagon Devices) deployed around a Cosmic Ray Neutron Sensor (CRNS). The two experimental sites are the GOR1 and MFC2 sub-catchments, where loamy and clayey soils are the predominant soil types, respectively. Therefore, these two sites are suitable for investigating the influence of soil texture on sensor readings. The GS3 sensors are usually operated with a customized calibration to determine soil water content from *K_b_* readings. However, it has been shown that the factory calibration is not accurate for soils with high clay content [21]. In addition, soil temperature effects on *K_b_* readings are widely ignored [23]. To broaden the scope of the study, we also considered the TEROS-12 sensor (METER Group Inc., Pullman, WA, USA), which is the successor of the GS3 sensor. To our knowledge, temperature-corrected calibrations for soils with different clay contents are still missing for GS3 and TEROS-12 sensors.

The main objective of this study was to evaluate a new multi-point calibration approach for soils with medium to high clay content to increase the accuracy of soil water content estimates influenced by temperature variations. The approach developed in this study was tested by comparing the spatial-average recalibrated GS3 data with independent area-wide soil water content observations from a CRNS. 

## 2. Materials and Methods

### 2.1. Environmental Setting and Site Instrumentation

A Critical Zone Observatory (CZO) was established in 2016 in the Upper Alento River Catchment within the TERENO (TERrestrial ENvironmental Observatories) long-term ecosystem infrastructure network [24]. The Upper Alento River Catchment is located in southern Italy, and the climate is sub-humid Mediterranean with hot, dry summers and mild, wet winters. Two experimental sub-catchments (MFC2 and GOR1) were instrumented in the Upper Alento River Catchment. These two sub-catchments reflect different hydrogeological, pedological, physiographic, and land-use features. MFC2 has an area of 8 ha and is representative of arable land with sparse cherry, walnut, and olive trees planted on the south-facing hillslopes with a gentle topography. The dominant soil texture classes in MFC2 are clay and clay loam (spatially averaged sand, silt, and clay contents are 21.6%, 40.5%, and 38.0%, respectively). GOR1 has an area of 18 ha and is located on an impervious and steep north-facing hillslope covered by dense mixed chestnut and oak woods. The predominant texture class in GOR1 is loam, with spatially averaged sand, silt, and clay contents of 40.7%, 37.4%, and 21.9%, respectively [25].

**Figure 1:**
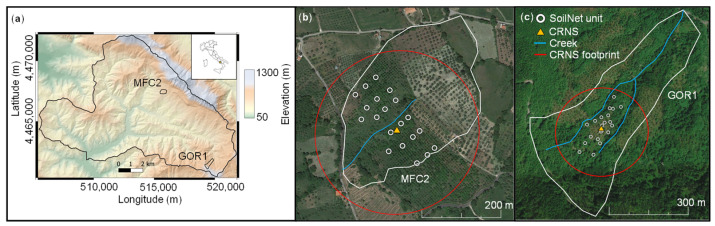
(**a**) Geographical position of the Upper Alento River Catchment (UARC) in Italy and position of MFC2 and GOR1 sites in UARC represented through a 5 m Digital Elevation Model (DEM), (**b**) location of the SoilNet unit devices, Cosmic Ray Neutron Sensor (CRNS) and its measurement footprint (red circle with a radius of 180 m) at MFC2 (clay soil), (**c**) location of the SoilNet unit devices, Cosmic Ray Neutron Sensor (CRNS) and its measurement footprint (red circle with a radius of 180 m) at GOR1 (loam soil).

A wireless sensor network (SoilNet, Forschungszentrum Juelich, Germany; [26]) was installed in both MFC2 and GOR1 controlling GS3 sensors at soil depths of 15 and 30 cm at 20 locations to monitor apparent dielectric bulk permittivity, *K_b_* (unitless), soil temperature, *T* (°C), and bulk electrical conductivity, *EC_b_* (mS cm^−1^) (Figure 1). The SoilNet stations were deployed around a stationary cosmic-ray neutron sensor (CRS2000/B by Hydroinnova LLC, Albuquerque, NM, USA), which was calibrated in a previous study [25]. A weather station was installed nearby each experimental site. Hourly and daily values of precipitation (*P*), minimum, mean, and maximum air temperature, relative humidity, wind speed, and net solar radiation have been measured since the installation in 2016. Daily values of crop-reference potential evapotranspiration (*ET*_0_) were calculated using the Penman–Monteith equation.

### 2.2. Laboratory Calibration Experiment

A laboratory calibration experiment was executed to evaluate the temperature dependency of GS3 and TEROS-12 sensors (METER Group Inc., Pullman, WA, USA). Both sensors generate a 70 MHz oscillating wave that charges the soil surrounding three 5.5-cm-long needles. The resulting measurement volume is about 160 cm^3^ for the GS3 and about 1010 cm^3^ for the TEROS-12 sensor. A ferrite core positioned on the TEROS-12 sensor cable helps relax any interferences in the electronic system. 

While the GS3 outputs the dielectric permittivity (*K_b_*) directly, the TEROS-12 sensor requires *RAW* values (sensor outputs depending on soil permittivity) to be converted into *K_b_* using the following equation:

(1) $${\;K}_{b}={\left(2.887\times 10^{-9}\times {RAW}^{3}-2.080\times 10^{-5}\times {RAW}^{2}+5.276\times RAW-43.39\right)}^{2}$$

To convert the *K_b_* outputs of the GS3 into soil water content (*θ*), the manufacturer provides the following third-order polynomial equation:

(2) $$\theta =5.89\times 10^{-6}K_{b}^{3}-7.62\times 10^{-4}K_{b}^{2}+3.67\times 10^{-2}K_{b}-7.53\times 10^{-2}$$

and the following equation to convert the *RAW* data of the TEROS-12 outputs into *θ*:

(3) $$\theta =3.879\times 10^{-4}RAW-0.6956$$

Note that *θ* refers to the volumetric water content (cm^3^ cm^−3^) defined as the volume of water within a given soil volume. To calibrate both sensor types by considering temperature effects, disturbed soil samples were collected from the MFC2 (clay soil) and GOR1 (loam soil) sites at soil depths between 15 cm and 30 cm in a representative position of the experimental site and transported to the laboratory. Soils were ground, passed through a 2 mm mesh sieve, and oven-dried at 105 °C for at least 24 h. Two soil samples per site were packed around a vertically oriented sensor in a 10-cm-tall and 14-cm-diameter plexiglass cylinder (with a volume of 1539 cm^3^) to achieve a target oven-dry soil bulk density (*ρ_b_* in g cm^−3^) corresponding to the spatial average value of each experimental site (*ρ_b_* =1.26 g cm^−3^ and *ρ_b_* =1.11 g cm^−3^ at MFC2 and GOR1, respectively). The diameter of the cylinder allows sufficient distance between the sensor and the wall to ensure that the sensing volume is within the soil material. A cheesecloth was used at the bottom end of the cylinder to prevent soil loss during sample handling. Soil samples were placed on a perforated ceramic plate and gradually saturated with deionized water, which was used to relax the impact of electrical conductivity on soil water content. After saturation, both cylinder ends were sealed with parafilm wax to avoid water loss due to evaporation. 

Subsequently, the samples were exposed to temperature variations while continuously monitoring changes in *K_b_*, *T*, and *EC_b_* every five minutes using a ZL6 datalogger (METER Group, Inc., Pullman, WA, USA). The imposed temperature variations ranged from 10 to 40 °C to represent the same variation observed in the field and were applied as follows: First, the samples were placed in a refrigerator for about 24 h or until thermal equilibrium was reached. In the next step, the soil samples were transferred into the oven at a temperature of 40 °C for about 24 h, or until thermal equilibrium was reached. After this, the seal at the top of the sample was removed to allow evaporation until the next target soil water content was reached. After this, the samples were sealed again, and the procedure described above was repeated. At the end of each experiment, each soil core was placed into the oven at a temperature of 105 °C for at least 24 h to determine oven-dry bulk density (*ρ_b_*) and *θ* gravimetrically. Each experiment was replicated twice using two different GS3 and TEROS-12 sensors, because a total of 80 GS3 sensors were deployed in the two experimental sites (MFC2 and GOR1). Sensor-to-sensor variability effects were thus ignored by crudely assuming limited sensor-to-sensor variability.

To interpret the experimental results, a linear regression equation that relates the square root of the apparent dielectric permittivity with the soil water content [27] was used:

(4) $$\theta =a\;\sqrt{K_{b}}+b$$

where *a* and *b* denote the slope and intercept of the regression function. Equation (4) represents a simplification of the physically based Complex Refraction Index Model (CRIM) with an additional term denoting bound water fraction [19,28] (Appendix A). Temperature effects were considered by assuming temperature-dependent slope and intercept values *a*(*T*) and *b*(*T*), which is consistent with expectations based on the CRIM model (Appendix A).

### 2.3. Evaluation Criteria to Test the Calibration and Validation Performance

To quantify the predictive accuracy of the calibration equations, two statistical performance indicators were used: the root mean square error (*RMSE*) and the coefficient of determination (*R*^2^). These indicators are defined as:

(5) $$RMSE=\sqrt{\frac{1}{n}\sum_{i}^{n}{\left(O_{i}-P_{i}\right)}^{2}}$$

and

(6) $$R^{2}=\frac{\sum_{i}^{n}{\left(O_{i}-P_{i}\right)}^{2}}{\sum_{i}^{n}{\left(O_{i}-\bar{O}\right)}^{2}}$$

where O, $\bar{O}$, and *P* are the observed, mean of observed, and predicted values of either soil permittivity or soil water content, respectively, *i* is the counter for data pairs, and *n* is the total number of data pairs. For an optimal prediction, values should be as low as possible for *RMSE* and as close as possible to 1 for *R*^2^. To qualitatively describe the calibration results, we follow the approach of Fares et al. (2011) [29], who classified the accuracy of capacitance sensors as good (*RMSE* < 0.01 cm^3^ cm^−3^), fair (0.01 ≤ *RMSE* < 0.05 cm^3^ cm^−3^), poor (0.05 ≤ *RMSE* < 0.10 cm^3^ cm^−3^), and very poor (*RMSE* ≥ 0.10 cm^3^ cm^−3^).

## 3. Results and Discussion

### 3.1. Relationship between Apparent Dielectric Permittivity, Temperature, and Soil Water Content

**Figure 2:**
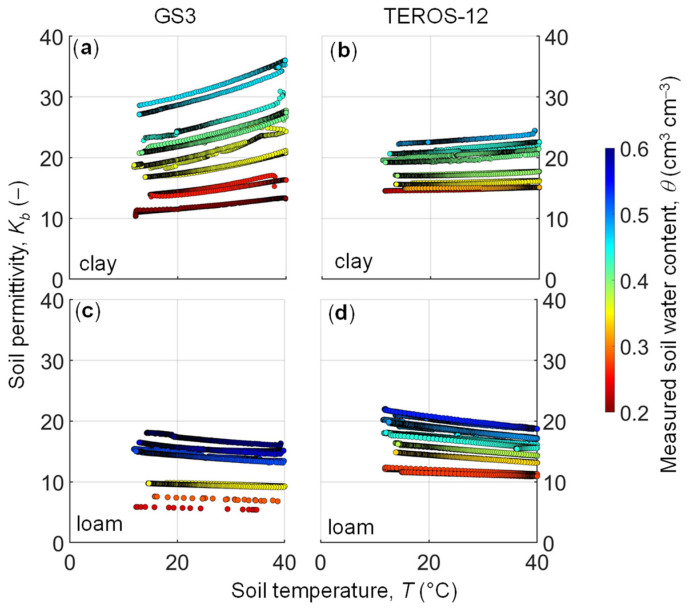
Relationship between soil apparent dielectric permittivity (*K_b_*) and temperature (*T*) in the soil samples collected in MFC2 (clay soil) by using (**a**) GS3 sensor, (**b**) TEROS-12 sensor, and in the soil samples collected in GOR1 (loamy soil) by using (**c**) GS3 sensor, (**d**) TEROS-12 sensor. Data points are color-coordinated by the soil water content that was measured gravimetrically.

A total of 7828 simultaneous measurements of *K_b_*, *T*, and *EC_b_* were recorded for both sensors and for each target soil water content. Figure 2 shows the relationship between soil permittivity and temperature at target soil water content values for the MFC2 (clay) and GOR1 (loam) sites using the GS3 and TEROS-12 sensors. The ZL6 datalogger does not automatically record permittivity data below 10, therefore *K_b_* data were taken manually under dry conditions. Some jumps in the permittivity recordings can be observed in the first soil water content steps (in wet conditions), which are due to abrupt movements during transport of the soil samples from the oven to the refrigerator and vice versa. In general, the highest sensitivity of the permittivity to soil temperature was observed near saturation. For the loamy soil (GOR1), soil permittivity decreases with increasing soil *T* (Figure 2c,d). This decrease is expected and related to the thermal response of the dielectric permittivity of water, which is well documented [28,30,31]. Similar decreasing trends have been reported for coarse and medium-textured soils [31,32]. In contrast, the *T*-*K_b_* relationships of the clay soil (MFC2) showed an increase in measured permittivity (GS3) or nearly independent soil permittivity (TEROS-12) with increasing temperature. Wraith and Or (1999) [30] used TDR to measure permittivity and soil water content at different temperatures for four soils with contrasting textures. In their study, the permittivity was inversely related to temperature for a loamy sand, while a silt loam, a clay, and a loam sand were characterized by a mixed behavior depending on soil wetness. This complicated dependence was attributed to the opposing effects of temperature on the free and bound water fractions. Molecules in free water rotate freely following an alternating electrical field, and permittivity decreases with increasing temperature. In contrast, the water molecules in bound water are attracted to the soil surface by adhesive, cohesive, and osmotic forces. The rotation of bound water molecules following an applied electrical field is restricted, resulting in less polarization compared with that of free water, and a low dielectric permittivity. This results in a thermodielectric response in the electromagnetic-based soil moisture measurement as the proportion of bound water decreases with increasing temperature [30].

Figure 2 shows different responses for the GS3 and TEROS-12 sensors, which is unexpected given the similarity in sensor design. According to manufacturer information, the measurement volume of the GS3 sensor is about six times smaller than that of the TEROS-12 sensor and consequently might be more susceptible to soil heterogeneity. Moreover, the lack of the ferrite core on the GS3 sensor cable might explain the sensitivity of permittivity to electrical signals disturbed by interferences in the electronic system (Figure 2a). Previous studies have shown that the sensor electronics can be affected by temperature changes, also leading to temperature effects in the sensor readings [10]. Therefore, additional differences in the responses of two sensor variants could be due to changes in the electronic parts or in the internal data processing, but, unfortunately, the manufacturer does not provide any information on this. 

### 3.2. Laboratory Calibration

**Figure 3:**
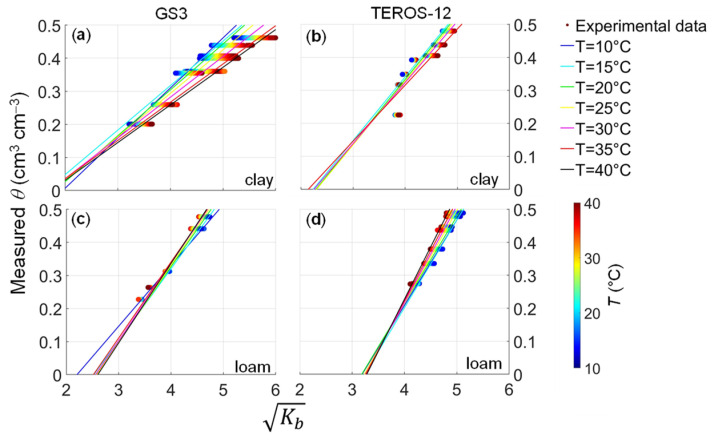
Relationship between the square root of apparent dielectric permittivity, $\sqrt{K_{b}}$, and soil water content, *θ*, measured gravimetrically by using the (**a**) GS3 sensor in the clay soil, (**b**) TEROS-12 sensor in the clay soil, (**c**) GS3 sensor in the loam soil, (**d**) TEROS-12 sensor in the loam soil. The circles are color-coordinated by soil temperature, *T*, and fitting curves (Equation (4)) are shown for different *T* (from 10 to 40 °C in 5 °C steps).

The relationship between the square root of apparent dielectric permittivity, $\sqrt{K_{b}}$ and soil water content, *θ* (from either GS3 or TEROS-12) was examined for different temperatures (Figure 3). Generally, the temperature effect changes with *θ* for both soils. When using the GS3 sensor in the clay soil at *θ* = 0.20 cm^3^ cm^−3^, $\sqrt{K_{b}}$ ranges between 3.21 and 3.66, while at *θ* = 0.46 cm^3^ cm^−3^ $\sqrt{K_{b}}$ ranges between 5.21 and 5.99 (Figure 3a). The temperature effect is less pronounced when using the TEROS-12 sensor in the clay soil, with $\sqrt{K_{b}}$ ranging between 3.81 and 3.91 at *θ* = 0.23 cm^3^ cm^−3^ and 4.71 and 4.92 at *θ* = 0.48 cm^3^ cm^−3^ (Figure 3b). In the loam soil, the difference between maximum and minimum $\sqrt{K_{b}}$ is 0.10 and 0.21 at the lowest (*θ* = 0.23 cm^3^ cm^−3^) and the highest (*θ* = 0.48 cm^3^ cm^−3^) *θ*, respectively, when using the GS3 sensor (Figure 3c). The difference is 0.09 and 0.35 at the lowest (*θ* = 0.27 cm^3^ cm^−3^) and the highest (*θ* = 0.54 cm^3^ cm^−3^) *θ*, respectively, when using the TEROS-12 sensor (Figure 3d).

Marked differences can be observed between the two soils when fitting the regression model (Equation (4)) to experimental data ($\sqrt{K_{b}}$-*θ*) for each 5 °C temperature step (Table 1). The prediction performance of the regression functions for the loam soil is diagnosed by *RMSE* values between 0.004 and 0.007 cm^3^ cm^−3^ when using the GS3 sensor, and between 0.002 and 0.011 cm^3^ cm^−3^ when using the TEROS-12 sensor. The *RMSE* values obtained for the clay soil ranged between 0.005 and 0.019 cm^3^ cm^−3^ when using the GS3 sensor, and between 0.025 and 0.031 cm^3^ cm^−3^ when using the TEROS-12 sensor. It should be noted that two temperature steps (10 °C and 40 °C) are missing for the TEROS-12 sensor applied to the clay soil for lack of experimental data. The *R*^2^ values are higher than 0.75 for all situations, which indicates a good fit quality (Table 1). 

**Figure 4:**
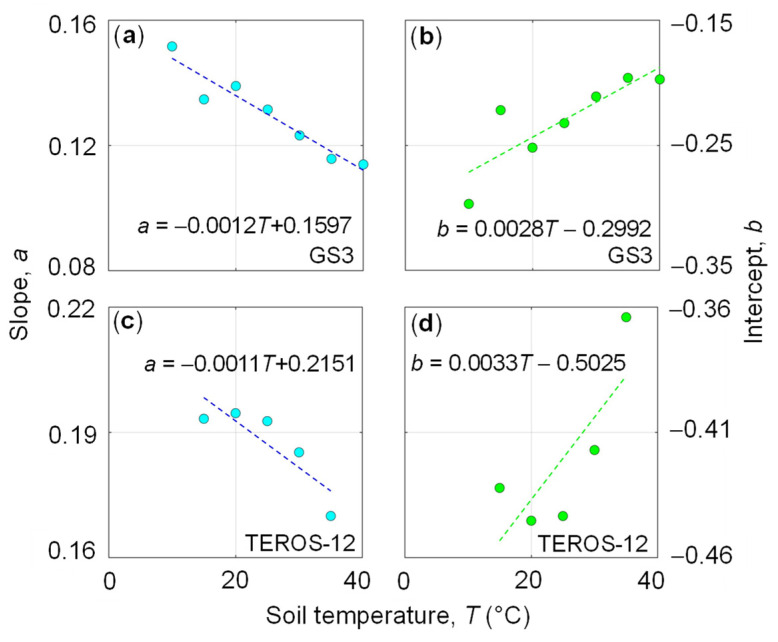
Relationship between temperature, *T* and (**a**) slope, *a* for GS3, (**b**) intercept, *b* for GS3, (**c**) slope, *a* for TEROS-12, (**d**) intercept, *b* for TEROS-12. All data refer to the clay soil (MFC2). The fitted coefficients *c* and *d* of Equation (7) for slope, *a* (blue dots) are reported in (**a**) (GS3) and (**c**) (TEROS-12) while the fitted coefficients *e* and *f* of Equation (8) for intercept, *b* (green dots) are reported in (**b**) (GS3) and (**d**) (TEROS-12).

**Figure 5:**
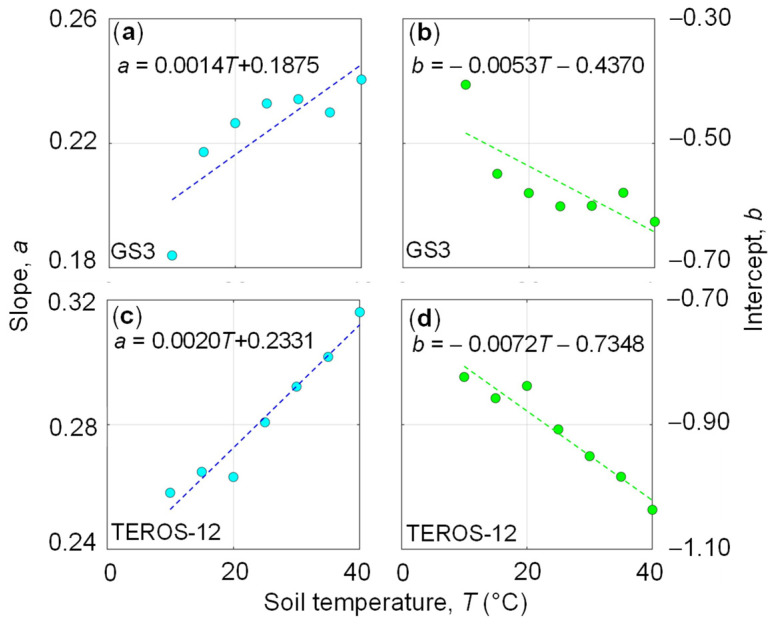
Relationship between temperature, *T* and (**a**) slope, *a* for GS3, (**b**) intercept, *b* for GS3, (**c**) slope, *a* for TEROS-12, (**d**) intercept, *b* for TEROS-12. All data refer to the loam soil (GOR1). The fitted coefficients *c* and *d* of Equation (7) for slope, *a* (blue dots) are reported in (**a**) (GS3) and (**c**) (TEROS-12) while the fitted coefficients *e* and *f* of Equation (8) for intercept, *b* (green dots) are reported in (**b**) (GS3) and (**d**) (TEROS-12).

In a next step, the dependence of the regression coefficients *a* and *b* on temperature was investigated for both the clay (Figure 4) and loam (Figure 5) soil. In general, the parameters changed substantially with temperature and showed different relationships with *T* for the clay and loam soil samples. For the clay soil, the *a* and *b* coefficients decreased and increased with temperature, respectively, for both sensor types (Figure 4). However, the weak relationship between the soil temperature and the regression coefficients (Figure 4c,d) for the TEROS-12 leads to inconsistent results. In contrast, the *a* and *b* coefficients changed in the opposite manner with increasing *T* for the loam soil (Figure 5). To describe this temperature dependence to some extent, we expressed the slope, *a* (Equation (4)) as a function of *T* by fitting the following regression line to *T*-*a* data:

(7) a=c T+d

where *c* and *d* are the slope and intercept, respectively. Similarly, the intercept, *b* (Equation (4)) was also described by the following linear regression:

(8) b=e T+f

where *e* and *f* are the slope and intercept, respectively. The regression coefficients (*c*, *d*, *e*, *f*) associated with the regression lines are reported in Figure 4 and Figure 5.

**Figure 6:**
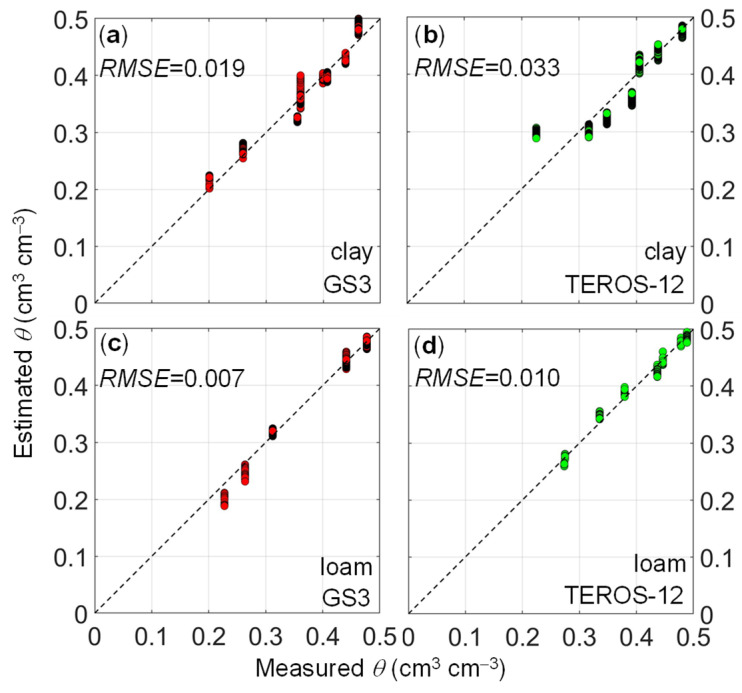
Comparison between soil water content measured using the thermogravimetric method and soil water content estimated using the temperature-corrected calibration equation for (**a**) clay soil with GS3 sensor (red dots), (**b**) clay soil with TEROS-12 sensor (green dots), (**c**) loam soil with GS3 sensor (red dots), (**d**) loam soil with TEROS-12 sensor (green dots). The diagonal dashed line depicts the 1:1 line.

The combination of Equations (7) and (8) with Equation (4) results in a temperature-corrected calibration, which was compared to the temperature-independent factory calibration (Table 2 and Figure 6). The *RMSE* and *R*^2^ values obtained from the temperature-corrected calibration as well as the temperature-independent factory calibration are reported in Table 2. The temperature-corrected calibration outperforms the factory calibration in all cases. The *RMSE* and *R*^2^ values obtained after calibration indicate good prediction performance, as visualized in Figure 6, where the measured and predicted soil water content match well [29]. Overestimation was observed in dry conditions for the TEROS-12 sensor in the clay soil (Figure 6b). For both soils, the prediction performance of the site-specific calibration using the GS3 sensor was better than the one obtained using the TEROS-12 sensor (Table 2). When using the GS3 sensor in the clay soil, the derived equations for *a*(*T*) and *b*(*T*) were able to well capture the spread of the $\sqrt{K_{b}}$-*θ* data (Figure 3a, *RMSE* = 0.019 cm^3^ cm^−3^ and *R*^2^ = 0.95). In contrast, the TEROS-12 sensor in the clay soil suffered from a lack of $\sqrt{K_{b}}$-*θ* data at temperature steps of 10 °C and 40 °C and misalignment of $\sqrt{K_{b}}$-*θ* data (Figure 3b), perhaps due to inexplicable experimental issues. Consequently, prediction performance was compromised in this case (*RMSE* = 0.033 cm^3^ cm^−3^ and *R*^2^ = 0.79). Good prediction performance was obtained when using both sensors in the loam soil. The *RMSE* obtained when using the GS3 sensor (*RMSE* = 0.007 cm^3^ cm^−3^) was lower than the one obtained from the TEROS-12 sensor (*RMSE* = 0.010 cm^3^ cm^−3^) due to the lower (Figure 3c) and higher (Figure 3d) scatter of $\sqrt{K_{b}}$-*θ* data, respectively. 

The temperature-independent factory calibration obtained acceptable to reasonable performance (*RMSE* = 0.052 cm^3^ cm^−3^ and *RMSE* = 0.065 cm^3^ cm^−3^ for the GS3 and TEROS-12 sensors, respectively) for the clay soil and reasonable to poor prediction (*RMSE* = 0.047 cm^3^ cm^−3^ and *RMSE* = 0.011 cm^3^ cm^−3^ for the GS3 and TEROS-12 sensors, respectively) for the loam soil. However, the high *R*^2^ values indicate good precision. Several previous studies have reported accuracies of calibration equations without temperature correction for different soil types. For example, Bhuiyan et al. (2020) [33] developed a second-order polynomial calibration equation for the GS3 sensor, which provided an *RMSE* of 0.086 cm^3^ cm^−3^ in a sandy soil. Ferrarezi et al. (2020) [34] also developed a soil-specific calibration for the GS3 sensor, which resulted in a *RMSE* of 0.054 cm^3^ cm^−3^ in five sandy soils in Florida (USA). Straten et al. (2014) [35] obtained an *RMSE* of 0.038 cm^3^ cm^−3^ in sandy soils of a field test facility designed to test the salt tolerance of crops under well-drained irrigated conditions. Son et al. (2017) [36] tested the GS3 sensor and obtained an *RMSE* of 0.028 cm^3^ cm^−3^ for a silt loam soil in South Korea. 

According to Fares et al. (2011) [29], we obtained fair (0.010 ≤ *RMSE* < 0.050 cm^3^ cm^−3^) to good (*RMSE* < 0.010 cm^3^ cm^−3^) accuracy for temperature-corrected calibration and poor (0.050 ≤ *RMSE* < 0.100 cm^3^ cm^−3^) to very poor (*RMSE* ≥ 0.100 cm^3^ cm^−3^) accuracy for default factory calibration. However, the prediction performance (*RMSE* = 0.033 cm^3^ cm^−3^) of temperature-corrected calibration for the TEROS-12 in the clay soil is still below the measurement accuracy (*RMSE* < 0.020 cm^3^ cm^−3^) recommended by Hignett and Evett (2008) [37] for research and agricultural applications. It should be noted that our study ignored the influence of temperature on the sensor electronics and the relationship between temperature and electrical conductivity. These aspects should be investigated in a follow-up analysis.

### 3.3. Comparison between Spatial-Average Temperature-Corrected and Area-Wide CRNS-Based Soil Water Content

**Figure 7:**
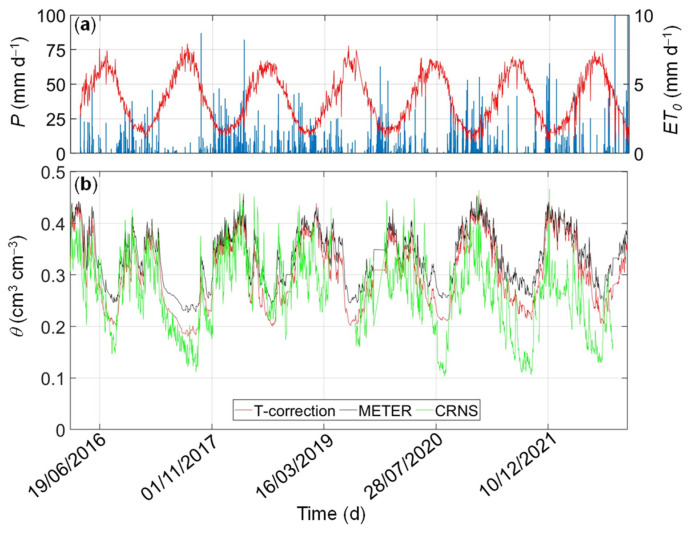
Daily values of (**a**) precipitation (*P*, blue bars) and crop-reference potential evapotranspiration (*ET*_0_, red line), (**b**) spatial-average soil water content at a soil depth of 15 cm were estimated by using temperature-corrected (*TCC*, red line) and factory (*FC*, black line) calibrations at MFC2 (clay soil) site. The area-wide soil water content measured by the Cosmic Ray Neutron Sensor (CRNS, green line) is shown for further comparison.

**Figure 8:**
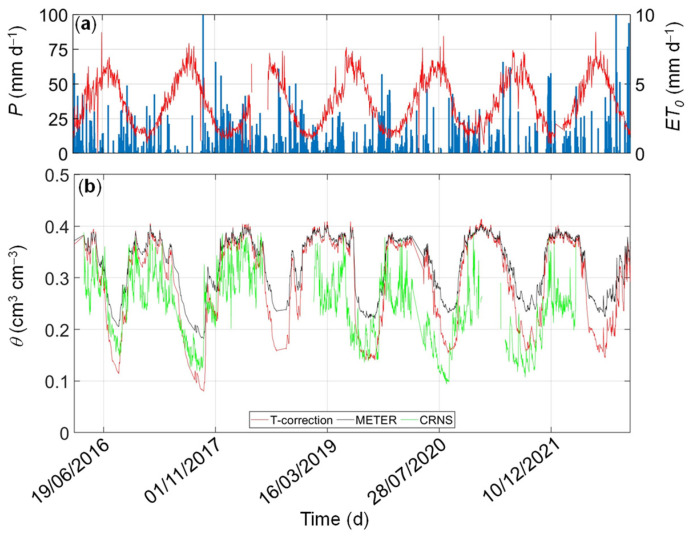
Daily values of (**a**) precipitation (*P*, blue bars) and crop-reference potential evapotranspiration (*ET*_0_, red line), (**b**) spatial-average soil water content at a soil depth of 15 cm were estimated by using temperature-corrected (*TCC*, red line) and factory (*FC*, black line) calibrations at GOR1 (loam soil) site. The area-wide soil water content measured by the Cosmic Ray Neutron Sensor (CRNS, green line) is shown for further comparison.

After establishing the temperature-dependent calibration in the laboratory, the feasibility of using such calibrations was evaluated in field studies. Unfortunately, it is not possible to rely on a field validation based on thermogravimetric measurements. Instead, the spatial average of the GS3-based soil water content data at the soil depth of 15 cm obtained with different calibrations was compared with the area-wide soil water content measured by a CRNS. The results are presented in Figure 7 for the clay soil and in Figure 8 for the loam soil. Frequent rainfall events during the wet season (usually from October to April) induced rapid responses in soil water content with spikes, followed by short periods of drainage. In contrast, long-term depletion induced by high evapotranspiration fluxes and sporadic rainfall events is observed during the dry season (from April to October). In general, soil water content data obtained with the CRNS and in-situ sensors agreed well in wet periods at the MFC2 site (clay soil) and showed deviations in prolonged dry periods (especially in the summers of 2017, 2020, 2021, and 2022). At the GOR1 site (loam soil), we observed an initial agreement between GS3-based and CRNS-based soil water content dynamics, which deteriorated in the last few years. Lack of CRNS data in some periods of time due to datalogger malfunctioning at GOR1 impedes further analysis. The observed deviations can be explained by the discrepancy in measurement depth between the two methods. The CRNS has a sampling depth of up to ~30 cm with a higher sensitivity to the first centimeters, whereas the GS3 sensors provide soil water content estimates at a soil depth of 15 cm.

**Figure 9:**
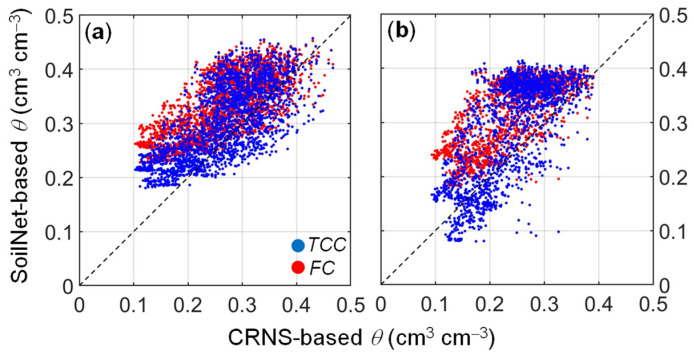
Comparison between area-wide CRNS-based and spatial-average SoilNet-based soil water content estimated by using temperature-corrected (*TCC*, blue circles) and factory (*FC*, red circles) calibrations at (**a**) MFC2 site (clay soil) and (**b**) GOR1 site (loam soil). The diagonal dashed line depicts the identity line (1:1 line).

The comparison between the two calibration methods highlights the impact of temperature on the prediction of soil water content. By assuming the temperature-corrected calibration method as the benchmark approach, the comparison between the two calibration equations revealed significant differences, especially during dry-down periods. In Figure 9, the CRNS- and SoilNet-based soil water content data are plotted against each other. The temperature-corrected calibration (*RMSE* = 0.067 cm^3^ cm^−3^ at MFC2 and *RMSE* = 0.082 cm^3^ cm^−3^ at GOR1) outperforms the temperature-independent factory calibration (*RMSE* = 0.086 cm^3^ cm^−3^ at MFC2 and *RMSE* = 0.096 cm^3^ cm^−3^ at GOR1). The bias of the factory calibration is evident through the misalignment of the data, which implies a slight overestimation under cold, wet conditions and a significant underestimation under warm, dry conditions.

## 4. Conclusions

Low-cost capacitance sensors are promising tools for monitoring soil water content at high temporal and spatial resolutions. Upon examining the data collected from the total of 80 GS3 sensors installed in the two experimental fields, the following question was raised: is the temperature-independent factory calibration reliable in the experimental areas characterized by soils with medium to high clay content and considerable temperature variations? To address the above question, a laboratory calibration strategy was developed to obtain site- and sensor-specific calibration equations that also accounted for soil temperature effects. However, only two GS3 and TEROS-12 sensors were used for each experiment, thus ignoring possible sensor-to-sensor variability effects that need to be investigated in a future study. The temperature-corrected calibration (*RMSE* from 0.007 cm^3^ cm^−3^ to 0.033 cm^3^ cm^−3^) outperformed the factory calibration (*RMSE* from 0.046 cm^3^ cm^−3^ to 0.110 cm^3^ cm^−3^). 

In addition, we evaluated the implications of using the factory calibration or a temperature-corrected calibration in two field studies with different soil characteristics. To this end, the GS3-based spatial-average soil water content was compared with the area-wide soil water content estimated using a cosmic-ray neutron sensor. The soil water content estimated by using temperature-corrected calibration outperformed the one based on the factory calibration, especially during the dry season of the Mediterranean climate. This study stressed the importance to resort to a site-specific laboratory experiment to properly convert apparent permittivity to soil water content under temperature variations. Therefore, the application of soil-specific temperature correction is highly recommended, especially if the capacitance sensors are installed in regions with pronounced seasonal variations in soil temperature.

## Figures and Tables

**Table 1:** Slope (*a*) and intercept (*b*) fitted in Equation (4), root mean square error (RMSE) and coefficient of determination (R^2^) for different soil temperatures (from 10 to 40 °C in 5 °C steps).

	*T*	*b*	*a*	*RMSE*	*R* ^2^
	°C			cm^3^ cm^−3^	
GS3	10	−0.297	0.152	0.005	0.995
	15	−0.222	0.135	0.019	0.932
	20	−0.252	0.139	0.016	0.953
	25	−0.232	0.131	0.018	0.946
	30	−0.211	0.123	0.019	0.943
	35	−0.196	0.116	0.017	0.940
	40	−0.197	0.114	0.018	0.960
TEROS-12	10				
	15	−0.432	0.193	0.029	0.765
	20	−0.445	0.195	0.025	0.888
	25	−0.443	0.193	0.029	0.844
	30	−0.417	0.185	0.031	0.803
	35	−0.364	0.170	0.029	0.776
	40				
GS3	10	−0.406	0.184	0.004	0.996
	15	−0.549	0.217	0.005	0.997
	20	−0.580	0.226	0.006	0.994
	25	−0.601	0.233	0.006	0.994
	30	−0.600	0.234	0.007	0.992
	35	−0.579	0.230	0.007	0.986
	40	−0.625	0.240	0.006	0.994
TEROS-12	10	−0.824	0.258	0.002	0.999
	15	−0.858	0.265	0.011	0.982
	20	−0.838	0.263	0.010	0.986
	25	−0.908	0.281	0.010	0.985
	30	−0.951	0.292	0.008	0.989
	35	−0.984	0.302	0.008	0.986
	40	−1.036	0.316	0.008	0.992

**Table 2:** Root mean square error (*RMSE*) and coefficient of determination (*R*^2^) of temperature-corrected (*TCC*) and factory (*FC*) calibration equations for clay and loam soil and for GS3 and TEROS-12 sensors.

Clay		*RMSE*	*R* ^2^
GS3	*TCC*	0.019	0.95
	*FC*	0.052	0.86
TEROS-12	*TCC*	0.033	0.79
	*FC*	0.065	0.79
Loam			
GS3	*TCC*	0.007	0.99
	*FC*	0.047	0.98
TEROS-12	*TCC*	0.010	0.99
	*FC*	0.111	0.87

## Data Availability

Data are available upon request.

## Appendix A

The Complex Refraction Index Model (CRIM) can be used to express the soil apparent permittivity, *K_b_* as [31]:

(A1) $$K_{b}={\left[\left(1-\eta \right)K_{s}^{\beta }+\left(\eta -\theta_{fw}-\theta_{bw}\right)K_{a}^{\beta }+\theta_{fw}K_{fw}^{\beta }+\theta_{bw}K_{bw}^{\beta }\right]}^{1/\beta }$$

where *K_fw_* and *K_bw_* are the permittivities of free and bound water fractions, respectively, *θ**_fw_* and *θ**_bw_* are the volume fractions of free and bound water, respectively, *η* is soil porosity, *β* is a shape factor (−1 < *β* < 1), and *K_s_*, *K_a_*, and *K_w_* are the permittivities of solids, air and water, respectively. By defining the total soil water content in the porous medium as *θ_tot_* = *θ_fw_* + *θ_bw_* and assuming that only *K_bw_*, *K_fw_*, and the different fractions of *θ* dependent on *T*, the following equation can be obtained by rearranging Equation (A1):

(A2) $$\theta_{tot}=\frac{K_{b}^{\beta }+\theta_{bw}(T)[K_{fw}^{\beta }(T)-K_{bw}^{\beta }(T)]-\left(1-\eta \right)K_{s}^{\beta }-\eta K_{a}^{\beta }}{K_{fw}^{\beta }(T)-K_{a}^{\beta }}$$

Here, the reduction in dielectric permittivity of free water with increasing temperature is well-documented [28]:

(A3) $$K_{fw}(T)=78.54\left[1-4.579\times 10^{-3}\left(T-25\right)+1.19\times 10^{-5}{\left(T-25\right)}^{2}-2.8\times 10^{-8}{\left(T-25\right)}^{3}\right]$$

The effect of temperature on the dielectric permittivity of bound water is unknown. By assuming *β* = 0.5 and *K_a_* = 1 in Equation (A2), the following expression can be obtained:

(A4) $$\theta_{tot}=\frac{1}{\sqrt{K_{fw}\left(T\right)}-1}\sqrt{K_{b}}+\frac{\theta_{bw}\left(T\right)\left[\sqrt{K_{fw}\left(T\right)}-\sqrt{K_{bw}(T)}\right]-\left(1-n\right)\sqrt{K_{s}}-n}{\sqrt{K_{fw}\left(T\right)}-1}$$

This equation can be simplified to:

(A5) $$\theta_{tot}=a(T)\sqrt{K_{b}}+b(T)$$
